# Prognostic effects of different nephroureterectomy techniques for upper urinary tract urothelial carcinoma: a network meta-analysis

**DOI:** 10.1186/s12885-025-13773-1

**Published:** 2025-02-28

**Authors:** Huan Wen, Yu Zhou, Lin Yang

**Affiliations:** Department of Urology, Bishan Hospital of Chongqing Medical University, Chongqing, 402760 China

**Keywords:** Upper urinary tract urothelial carcinoma (UTUC), Nephroureterectomy (NU), Cancer-specific survival (CSS), Overall survival (OS)

## Abstract

**Background:**

Upper urinary tract urothelial carcinoma (UTUC) is an aggressive malignant tumor, with surgical intervention as the primary treatment. This study evaluates the prognostic effects of laparoscopic radical nephroureterectomy (LNU), open radical nephroureterectomy (ONU), robot-assisted laparoscopic radical nephroureterectomy (Robotic LNU), and hand-assisted laparoscopic radical nephroureterectomy (Hand LNU) in the treatment of UTUC through a network meta-analysis.

**Methods:**

A systematic search of the PubMed, EMBASE, Cochrane, and Web of Science databases was conducted for randomized controlled trials meeting the criteria from database inception until April 2024. Bayesian network meta-analysis was performed to compare the effects of each surgical method on overall survival (OS), cancer-specific survival (CSS), and overall recurrence rate (ORE).

**Results:**

Seventeen randomized controlled trials were included in this network meta-analysis. The results indicated that LNU significantly improved CSS compared to ONU [HR = 0.81, 95%CI= (0.7, 0.93)], while there were no significant differences between Hand LNU and Robotic LNU compared to ONU. Among minimally invasive surgeries, Hand LNU significantly shortened CSS compared to LNU [HR = 1.49, 95%CI=(1.1, 2.03)]. Regarding ORE, no significant differences were found between LNU, Hand LNU, and Robotic LNU compared to ONU, although LNU had a higher recurrence rate than Robotic LNU [HR = 1.705, 95%CI=(1.007, 3.001)]. For OS, both LNU [HR = 0.84, 95%CI=(0.75, 0.94)] and Robotic LNU [HR = 0.81, 95%CI=(0.68, 0.96)] were significantly better than ONU, whereas Hand LNU significantly shortened OS compared to LNU and Robotic LNU. There were no significant differences in progression-free survival (PFS) between LNU and ONU. The cumulative efficacy ranking indicated that Robotic LNU ranked highest for ORE and OS, while LNU ranked first for CSS.

**Conclusion:**

Robotic LNU demonstrates advantages in prolonging OS and reducing recurrence rates, while LNU excels in improving CSS. Although Hand LNU shows suboptimal effects in some comparisons, it remains valuable, and surgical choices should be based on individualized needs.

## Introduction

Upper tract urothelial carcinoma (UTUC), recognized as a highly aggressive malignant tumor, primarily occurs in the renal pelvis and ureter. Although UTUC has a low incidence, it is no longer considered a “rare” tumor with the publication of relevant clinical guidelines. It is defined as the malignant transformation of urothelial cells lining the urinary tract from the renal calyces to the ureteral orifice, accounting for 5–10% of all urothelial cancers [1, 2]. Despite its relatively low incidence, UTUC typically has a poor prognosis, with a five-year survival rate for advanced UTUC patients significantly lower than that of other cancer types [3, 4]. The mechanisms underlying its occurrence are not yet fully understood, however, studies suggest that a combination of environmental factors, genetic predispositions, and lifestyle choices may play a crucial role in triggering UTUC [5]. For instance, prolonged exposure to harmful chemicals, smoking, and certain genetic mutations have been closely associated with the development of UTUC. Due to the subtlety of early symptoms, many patients are diagnosed at an advanced stage, complicating treatment.

Radical nephroureterectomy (NU) is the gold standard surgical treatment for high-risk, non-metastatic UTUC [6]. Given that open nephroureterectomy (ONU) can provide long-term local control of the disease and improve patient survival rates, it has become the most commonly used surgical approach for treating high-risk upper tract urothelial carcinoma. However, it may be associated with significant morbidity [7]. The first laparoscopic nephroureterectomy was performed in 1993 [8], and following decades of technological advancement, minimally invasive surgical (MIS) techniques, including hand-assisted laparoscopic nephroureterectomy (HandLNU), laparoscopic nephroureterectomy (LNU), and robotic-assisted laparoscopic nephroureterectomy (RoboticLNU), have been introduced as alternatives to ONU and are widely accepted for the treatment of UTUC.

This study aims to conduct a systematic review and network meta-analysis to compare the overall survival (OS), cancer-specific survival (CSS), overall recurrence rate (ORE), and progression-free survival (PFS) among patients with UTUC undergoing laparoscopic radical LNU, ONU, RoboticLNU, and HandLNU.

## Methods

We performed a systematic review based on the protocol registered under CRDxxx, following the Preferred Reporting Items for Systematic Reviews and Meta-Analyses (PRISMA) statement [9].

### Inclusion and exclusion criteria

Based on the definitions of participants, exposure, comparison, outcomes, and study types (PECOS) from the Cochrane Handbook for Systematic Reviews, the following inclusion and exclusion criteria were established.

#### Inclusion criteria

(1) Population (P): Adult patients clinically diagnosed with upper tract urothelial carcinoma.

(2) Exposure (E)/Comparison (C): Laparoscopic nephroureterectomy (LNU), open nephroureterectomy (ONU), robotic-assisted laparoscopic nephroureterectomy (Robotic LNU), and hand-assisted laparoscopic nephroureterectomy (Hand LNU).

(3) Outcomes (O): Overall Survival (OS): Refers to the time from the start of treatment until death, regardless of the cause of death; Cancer-Specific Survival (CSS): Refers to the time from the start of treatment until death due to cancer-related causes. It excludes deaths from other causes (e.g., heart disease), focusing only on mortality directly related to cancer; Overall Recurrence Rate (ORE): Refers to the proportion of patients whose cancer recurs after treatment, typically within a specified period. This includes both local recurrences and distant metastases; Progression-Free Survival (PFS): Refers to the length of time during and after treatment that a patient lives without the cancer worsening, progressing, or spreading.

(4) Study Types (S): Cohort studies, randomized controlled trials, or case-control studies.

#### Exclusion criteria

(1) Meta-analyses, reviews, systematic evaluations, expert consensus, in vitro studies, animal experiments, case reports, letters, and replies.

(2) Data that are evidently incorrect or missing, and for which the corresponding author cannot be contacted.

### Search strategy

We systematically searched PubMed, Embase, Cochrane Library, and Web of Science for relevant literature from the inception of each database until April 27, 2024. Keywords included “Upper Tract Urothelial Carcinoma,” “Nephroureterectomy,” and “Robotic Surgical Procedures,” with no restrictions on region or language. Supplemental Material 1 details the search strategies for each database.

### Literature screening and data extraction

Two researchers rigorously screened articles and extracted data according to the inclusion and exclusion criteria, with cross-checking of results. In case of disagreements, a third researcher was involved in the discussion to reach a conclusion. The two researchers independently extracted data based on a pre-designed checklist, including (1) basic characteristics of the studies, such as authors, publication year, country, patient source, gender, and sample size; (2) key elements for bias risk assessment; and (3) outcome measures. After data extraction, any discrepancies were resolved through discussion, or a third party was consulted if necessary.

### Bias risk assessment of included studies

Two reviewers independently evaluated the methodological quality of each included study using the Newcastle-Ottawa Scale (NOS), which comprises three major modules and eight items [10]. The evaluation covered three main aspects: selection of the study population (0–4), comparability between groups (0–2), and outcome measurement (0–3). A total score of 6 or higher was considered high-quality research. Any disagreements were resolved through discussion or arbitration by a third party if necessary.

### Statistical analysis

For binary variables, risk ratios (RR) with corresponding 95% confidence intervals (CIs) were used. For survival metrics such as OS, PFS, and CSS, hazard ratios (HR) and 95% confidence intervals (95% CI) were directly extracted from the included articles regarding the impact of surgical methods on prognostic indicators. If multiple estimates were reported in the same article, we selected the multivariable analysis results adjusted for confounding factors. Due to heterogeneity among studies, a Bayesian random effects model was applied for multiple comparisons of different nephroureterectomy techniques on the prognosis and recurrence of patients with upper tract urothelial carcinoma. Markov chain Monte Carlo methods were employed for modeling, with four Markov chains running simultaneously, setting the number of annealing iterations to 20,000 and completing the modeling after 50,000 simulation iterations. The Deviance Information Criterion (DIC) was utilized to compare model fit and overall consistency. In cases of closed-loop networks, the node-splitting method was further applied for local consistency analysis. Furthermore, efficacy rankings for each intervention were generated, along with league tables to compare effect differences among various interventions. All analyses were performed using Stata 15.1 and R software (VER 4.2.1).

## Results

### Systematic search results

**Fig. 1:**
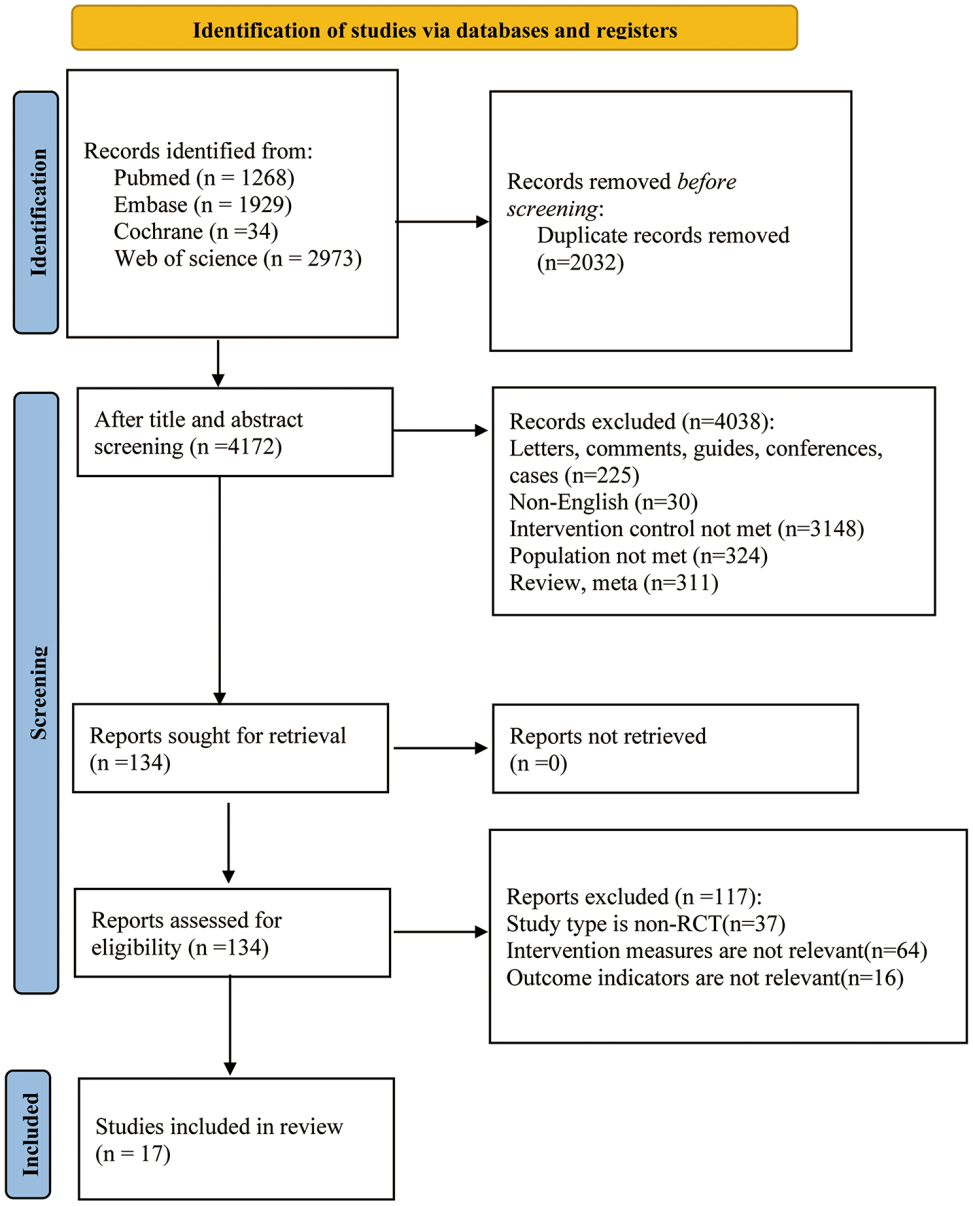
Literature screening flowchart

A total of 6,204 articles were retrieved from the databases. After removing 2,032 duplicates, 4,172 studies remained. By excluding 4,038 articles based on irrelevance in titles and abstracts, we were left with 134 articles. Upon reviewing the full texts, 117 articles that did not meet the inclusion criteria were discarded. Ultimately, 17 randomized controlled trials were included (Fig. 1).

### Basic information of included studies

**Fig. 2:**
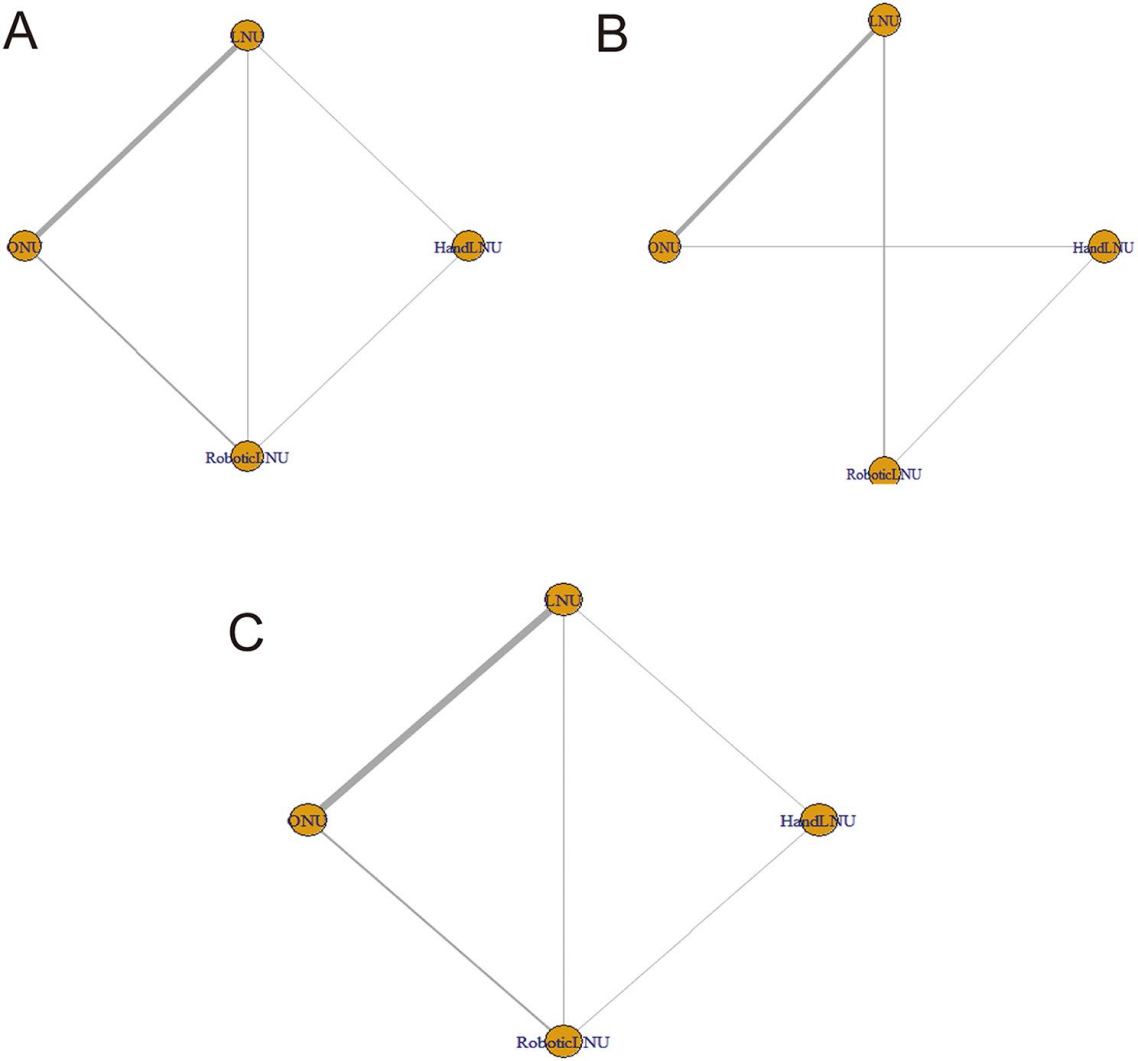
Network diagram **A**. CSS network diagram R language. **B**. OR network diagram R language. **C**. OS network diagram R language

The network meta-analysis incorporated 11,989 patients with UTUC from the 17 studies. This analysis encompassed four surgical approaches: LNU (4,643 patients), ONU (5,587 patients), robotic-assisted laparoscopic nephroureterectomy (Robotic LNU, 782 patients), and hand-assisted laparoscopic nephroureterectomy (Hand LNU, 977 patients). Table 1 presents the fundamental characteristics of each trial, while Fig. 2 illustrates the network diagram showing the outcomes associated with different interventions. Detailed results of the quality assessment based on the Newcastle-Ottawa Scale (NOS) are provided in Supplemental Material 2, with all studies scoring above 6, categorizing them as high-quality research.

**Table 1:** Baseline characteristics of included studies

Study	Year	Country	Study type	Data Resource	Patients	Treatment/Sample size				Total Sample	Male/Female	Outcomes
Qing’ao Cui	2022	China	retrospective	Single center	UTUC	/	/	LNU/51	RoboticLNU /19	70	40/30	ORE
Morgan Rouprêt	2007	France	retrospective	Single center	UTUC	/	ONU/26	LNU/20	/	46	34/12	ORE
Adrian S. Fairey	2012	Canada	retrospective	Multi center	UTUC	/	ONU/403	LNU/446	/	849	542/307	PFS, OS
Jun Miyazaki	2016	Japan	retrospective	Multi center	UTUC	/	ONU/527	LNU/222	/	749	504/245	ORE
Shiudong Chung	2007	China	retrospective	Single center	UTUC	HandLNU /39	ONU/36	/	/	75	35/40	ORE
Jian-Ye Liu	2017	China	retrospective	Multi center	UTUC	/	ONU/213	LNU/52	/	265	198/67	CSS, PFS, OS, ORE
Ching-Chia Li	2021	China	retrospective	Multi center	UTUC	HandLNU /741	/	LNU/458	RoboticLNU /141	1,340	578/762	CSS, OS
Che-Yuan Hu	2015	China	retrospective	Single center	UTUC	HandLNU /197	/	/	RoboticLNU /18	215	109/106	ORE
Vivek Vasudeo	2023	India	retrospective	Single center	UTUC	/	/	LNU/26	RoboticLNU /37	63	51/12	ORE
Simone Morselli	2021	Italy	retrospective	Multi center	UTUC	/	ONU/60	LNU/47	/	107	60/47	ORE
Sung Han Kim	2019	Korea	retrospective	Multi center	UTUC	/	ONU/638	LNU/638	/	1276	941/335	CSS, PFS, OS
Hyung Suk Kim	2016	Korea	retrospective	Single center	UTUC	/	ONU/271	LNU/100	/	371	287/84	CSS, OS
Koichi Kido	2018	Japan	retrospective	Multi center	UTUC	/	ONU/351	LNU/75	/	426	290/136	CSS, OS
Matthew B. Clements	2018	USA	retrospective	Single center	UTUC	/	ONU/1862	LNU/1624	RoboticLNU / 315	3801	2182/1619	CSS, OS
Tae Heon Kim	2019	Korea	retrospective	Multi center	UTUC	/	ONU/906	LNU/615	/	1521	1127/394	CSS, PFS, OS
Nico C. Grossmann	2023	Austria	retrospective	Multi center	UTUC	/	ONU/252	LNU/252	RoboticLNU /252	756	505/251	CSS, OS
Benedikt Hoeh	2023	Germany	retrospective	Single center	UTUC	/	ONU/42	LNU/17	/	59	41/18	ORE

UTUC: Upper Urinary Tract Urothelial Carcinoma; Hand LNU: Hand-Assisted Laparoscopic Radical Nephroureterectomy; ONU: Open Radical Nephroureterectomy; LNU: Laparoscopic Radical Nephroureterectomy; Robotic LNU: Robot-Assisted Laparoscopic Radical Nephroureterectomy; OS: Overall Survival; ORE: Overall Recurrence Rate; PFS: Progression-Free Survival; CSS: Cancer-Specific Survival

### Network meta-analysis

#### Cancer-specific survival

Eight studies [11–18] reported on CSS in patients with UTUC following surgical intervention. The NMA indicated a statistically significant improvement in CSS after LNU [HR = 0.81, 95% CI= (0.7, 0.93)] compared to ONU. However, no significant differences were observed for hand-assisted Hand LNU [HR = 1.21, 95% CI= (0.87, 1.68)] and Robotic LNU [HR = 0.86, 95% CI= (0.66, 1.12)] when compared to ONU. Among minimally invasive procedures, Hand LNU [HR = 1.49, 95% CI= (1.1, 2.03)] was associated with a reduced CSS relative to LNU (Table 2).

**Table 2:** CSS table

HandLNU			
1.49 (1.1, 2.03)	LNU		
1.4 (0.98, 2)	0.94 (0.72, 1.23)	RoboticLNU	
1.21 (0.87, 1.68)	0.81 (0.7, 0.93)	0.86 (0.66, 1.12)	ONU

#### Overall recurrence

Nine studies [15, 19–26] provided data on the ORE for UTUC patients after surgical intervention. The overall meta-analysis revealed no statistically significant differences among Hand LNU [RR = 0.935, 95% CI=(0.543, 1.615)], LNU [RR = 0.976, 95% CI=(0.745, 1.264)], and Robotic LNU [RR = 0.571, 95% CI=(0.315, 1)] compared to ONU. However, pairwise comparisons among minimally invasive surgeries indicated that LNU [RR = 1.705, 95% CI= (1.007, 3.001)] resulted in a statistically significant increase in overall recurrence compared to Robotic LNU (Table 3).

**Table 3:** ORE table

HandLNU			
0.958 (0.545, 1.699)	LNU		
1.628 (0.883, 3.176)	1.705 (1.007, 3.001)	RoboticLNU	
0.935 (0.543, 1.615)	0.976 (0.745, 1.264)	0.571 (0.315, 1)	ONU

#### Overall survival (OS)

Nine studies [11–18, 27] provided data on OS for patients with UTUC following surgical intervention. The meta-analysis demonstrated a significant increase in OS with LNU [HR = 0.84, 95% CI= (0.75, 0.94)] and Robotic LNU [HR = 0.81, 95% CI= (0.68, 0.96)] compared to ONU. In pairwise comparisons among minimally invasive techniques, HandLNU was associated with a significantly shorter OS compared to both LNU [HR = 1.39, 95% CI= (1.1, 1.74)] and Robotic LNU [HR = 1.44, 95% CI= (1.1, 1.88)] (Table 4).

**Table 4:** OS table

HandLNU			
1.39 (1.1, 1.74)	LNU		
1.44 (1.1, 1.88)	1.04 (0.87, 1.24)	RoboticLNU	
1.16 (0.91, 1.49)	0.84 (0.75, 0.94)	0.81 (0.68, 0.96)	ONU

### Ranking results

**Fig. 3:**
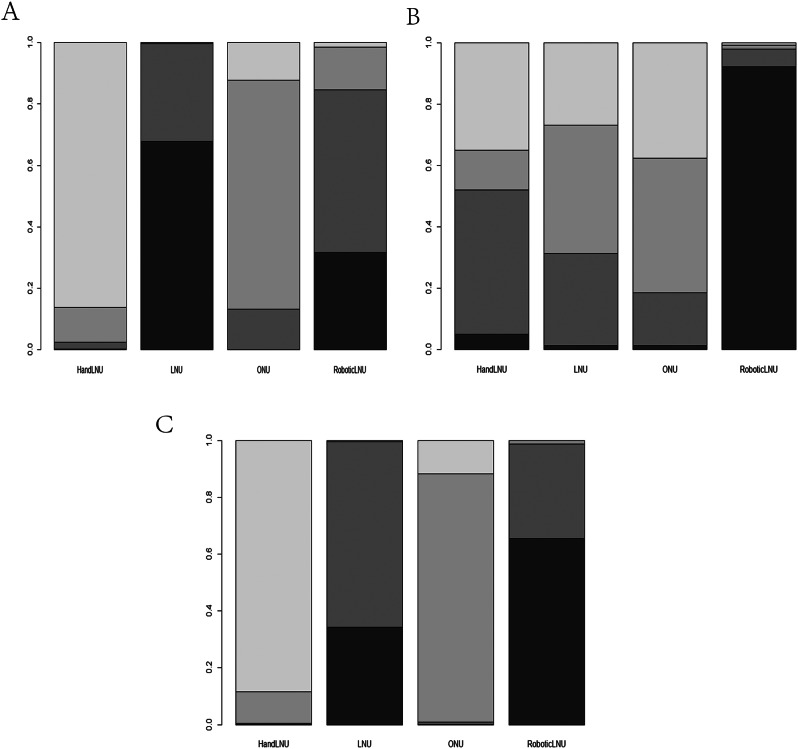
Sort diagram **A**. CSS Sort diagram. **B**. OR Sort diagram. **C**. S OS Sort diagram

The cumulative efficacy ranking chart indicated that Robotic LNU ranked highest for ORE and OS, while LNU held the top position for CSS (Fig. 3 & Supplemental Material 3).

### Inconsistency

The analysis using the DIC and the node-splitting method revealed no statistical inconsistency (Supplemental Material 2).

### Pairwise analysis

**Fig. 4:**
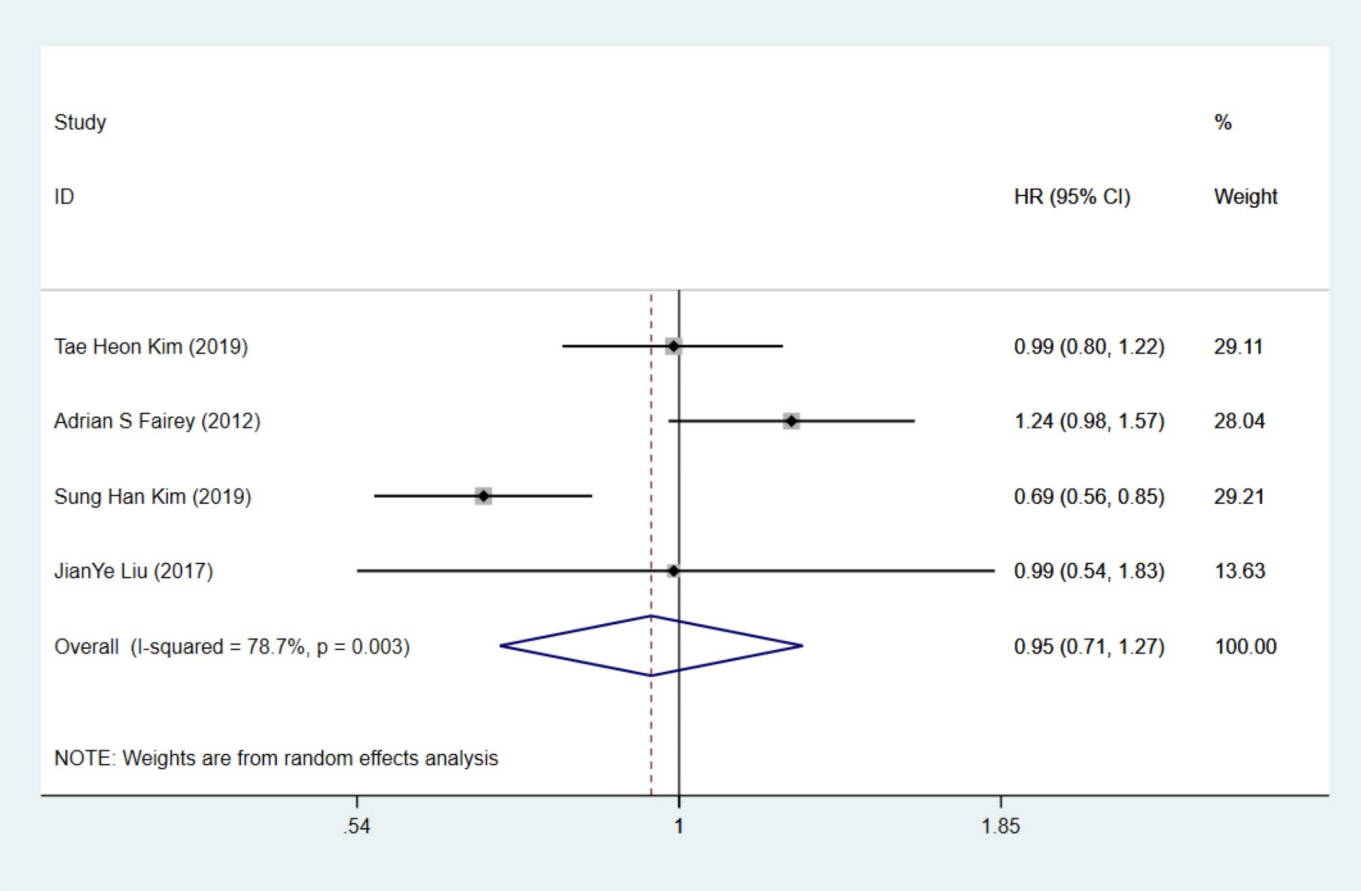
Forest map

Only four studies reported on PFS for UTUC patients undergoing LNU, leading us to conduct a pairwise analysis. The heterogeneity test indicated I²=78.7%, necessitating the use of a random effects model. The meta-analysis results showed no statistically significant difference between LNU and ONU [HR = 0.95, 95% CI= (0.71, 1.27), *P* > 0.05] (Fig. 4).

## Discussions

This network meta-analysis compares the prognostic outcomes of various nephroureterectomy techniques (LNU, ONU, Robotic LNU, HandLNU) in the treatment of UTUC. By synthesizing data from 17 cohorts, we identified differences among these surgical methods in terms of CSS, OS, ORR, and PFS. These findings provide critical insights for clinicians in selecting the most appropriate surgical approach.

Firstly, the analysis reveals that LNU excels in enhancing CSS. Compared to open surgery, LNU significantly improves cancer-specific survival rates, a result consistent with previous literature [28], likely due to the minimally invasive nature of LNU, which offers faster recovery while maintaining effective tumor control [29]. However, LNU is associated with a higher recurrence rate compared to Robotic LNU, suggesting that LNU may be less effective in controlling the risk of recurrence. This difference may stem from the complexity and precision required during surgery, where robotic technology offers a refined approach to narrow anatomical structures, reducing the likelihood of residual tumors and recurrence [30].

Robotic LNU demonstrated superior prognostic outcomes in this study, ranking first in improving OS and reducing ORR. This highlights that with advancements in robotic technology, Robotic LNU’s advantages in precision, stability, and enhanced visualization provide a significant edge in UTUC treatment [31]. Although the high cost and equipment requirements have limited its widespread adoption, the increasing availability and reduced costs of robotic surgery may position Robotic LNU as the preferred approach for UTUC treatment in the future.

HandLNU offers a novel minimally invasive alternative for UTUC patients [32]. It employs a unique method combining the benefits of open and laparoscopic surgery, along with optimal specimen retrieval, thereby preserving oncological principles traditionally adhered to in open surgery [33]. Nevertheless, the results of this study indicate that HandLNU is suboptimal compared to other minimally invasive techniques. It significantly shortens CSS and OS compared to LNU and Robotic LNU, possibly due to its operative limitations. Although hand-assisted laparoscopic techniques provide surgeons with better tactile feedback, their limited precision and surgical field may compromise efficacy in complex cases. Furthermore, prior studies suggest that HandLNU may be inferior to LNU or ONU in terms of progression-free survival and bladder recurrence-free survival [34, 35]. However, HandLNU remains a potential option, particularly in resource-limited settings or for specific patient populations where it may retain some clinical value.

It is noteworthy that ONU, as a traditional surgical method, performs relatively poorly in terms of CSS and OS, likely due to higher postoperative complications and longer recovery periods. Although ONU was once the mainstay for UTUC treatment, the rise of minimally invasive techniques has diminished its prognostic advantage. This finding further underscores the significance of minimally invasive surgery, particularly in improving patient quality of life and postoperative recovery, where minimally invasive procedures clearly demonstrate superior benefits.

Although this study provides valuable comparative evidence on the prognostic outcomes of different surgical methods for the treatment of UTUC, there are certain limitations. First, the number of studies included is relatively small, particularly regarding Robotic LNU and HandLNU, which may affect the comprehensive assessment of the prognostic effects of these techniques. Secondly, the differences in baseline patient characteristics, surgical techniques, and postoperative management strategies across studies introduced heterogeneity that could influence the results. For instance, regarding the patient population for robotic-assisted surgery, we acknowledge that robotic surgery is typically applied to a more strictly selected group of patients, such as those who are younger, in better physical condition, and with early-stage tumors. These factors may result in robotic surgery demonstrating significant advantages in CSS and OS. Therefore, the outcomes of robotic surgery may to some extent reflect the patient selection criteria rather than the independent advantage of the technology itself. Although we employed Bayesian network meta-analysis to mitigate biases from these differences, it is difficult to entirely eliminate their impact. Thirdly, we also note that the influence of vesical cuff management (intravesical, extravesical, or endoscopic) on bladder recurrence has not been fully explored in the current analysis. The management of the vesical cuff should be tailored based on the patient’s tumor stage and specific condition. Although this study did not specifically investigate the impact of different vesical cuff management techniques on bladder recurrence, future research should focus on this aspect, evaluating the clinical outcomes of different cuff excision methods to help refine treatment strategies and improve patient prognosis. Lastly, while we assessed major prognostic indicators such as CSS, OS, and ORR, other potential factors influencing prognosis, such as surgical complications, quality of life, and economic costs, were not fully considered. Future research should focus on large-scale, prospective randomized controlled trials to better evaluate the long-term efficacy and indications of different surgical methods, while standardizing the reporting of prognostic indicators to improve the reliability and generalizability of the analysis results.

It is worth noting that, in current treatment trends, kidney-sparing treatment has become an increasingly popular option for UTUC patients, especially in cases where the tumor is well-localized and does not invade the renal parenchyma. According to the latest guidelines from the American Urological Association (AUA), kidney-sparing surgery is recommended for patients who meet specific criteria, aiming to preserve renal function and reduce the need for dialysis [36]. However, for advanced disease or high-risk patients, radical nephrectomy remains the preferred treatment. Therefore, future research should further evaluate the efficacy of kidney-sparing surgery in different patient populations, particularly in terms of long-term prognosis and quality of life.

## Conclusions

This network meta-analysis comprehensively compared the prognostic outcomes of different surgical approaches for the treatment of UTUC, including LNU, ONU, robotic LNU, and HandLNU. The results indicate that robotic LNU offers significant advantages in extending OS and reducing OER. LNU demonstrated superior performance in improving CSS, underlining its effectiveness in long-term cancer control. Although HandLNU did not perform as well as LNU in certain outcomes, it remains a viable option in resource-constrained settings. In contrast, traditional ONU showed inferior prognostic outcomes compared to more advanced techniques. Ultimately, the choice of surgical approach should be tailored to the patient’s specific circumstances, surgical complexity, and clinical needs to optimize treatment outcomes.

## Data Availability

The datasets used or analysed during the current study are available from the corresponding author on reasonable request.

## References

[1] O’Sullivan NJ, Naughton A, Temperley HC, Casey RG. Robotic-assisted versus laparoscopic nephroureterectomy; a systematic review and meta-analysis. BJUI Compass. 2023;4(3):246–55. 37025468 10.1002/bco2.208 PMC10071076

[2] Azizi M, Cheriyan SK, Peyton CC, Foerster B, Shariat SF, Spiess PE. Optimal management of upper tract urothelial carcinoma: an unmet need. Curr Treat Options Oncol. 2019;20(5):40. 30937554 10.1007/s11864-019-0637-2

[3] Margulis V, Shariat SF, Matin SF, Kamat AM, Zigeuner R, Kikuchi E, Lotan Y, Weizer A, Raman JD, Wood CG. Outcomes of radical nephroureterectomy: a series from the upper tract urothelial carcinoma collaboration. Cancer. 2009;115(6):1224–33. 19156917 10.1002/cncr.24135

[4] Abouassaly R, Alibhai SMH, Shah N, Timilshina N, Fleshner N, Finelli A. Troubling outcomes from population-level analysis of surgery for upper tract urothelial carcinoma. Urology. 2010;76(4):895–901. 20646743 10.1016/j.urology.2010.04.020

[5] Soria F, Shariat SF, Lerner SP, Fritsche H-M, Rink M, Kassouf W, Spiess PE, Lotan Y, Ye D, Fernández MI, et al. Epidemiology, diagnosis, preoperative evaluation and prognostic assessment of upper-tract urothelial carcinoma (UTUC). World J Urol. 2017;35(3):379–87. 27604375 10.1007/s00345-016-1928-x

[6] Rouprêt M, Babjuk M, Burger M, Capoun O, Cohen D, Compérat EM, Cowan NC, Dominguez-Escrig JL, Gontero P, Hugh Mostafid A, et al. European association of urology guidelines on upper urinary tract urothelial carcinoma: 2020 update. Eur Urol. 2021;79(1):62–79. 32593530 10.1016/j.eururo.2020.05.042

[7] Rouprêt M, Babjuk M, Compérat E, Zigeuner R, Sylvester RJ, Burger M, Cowan NC, Böhle A, Van Rhijn BWG, Kaasinen E, et al. European association of urology guidelines on upper urinary tract urothelial cell carcinoma: 2015 update. Eur Urol. 2015;68(5):868–79. 26188393 10.1016/j.eururo.2015.06.044

[8] Rassweiler JJ, Henkel TO, Potempa DM, Coptcoat M, Alken P. The technique of transperitoneal laparoscopic nephrectomy, adrenalectomy and nephroureterectomy. Eur Urol. 1993;23(4):425–30. 8335045 10.1159/000474647

[9] Moher D, Liberati A, Tetzlaff J, Altman DG. Preferred reporting items for systematic reviews and meta-analyses: the PRISMA statement. BMJ. 2009;339:b2535. 19622551 10.1136/bmj.b2535 PMC2714657

[10] Stang A. Critical evaluation of the Newcastle-Ottawa scale for the assessment of the quality of nonrandomized studies in meta-analyses. Eur J Epidemiol. 2010;25(9):603–5. 20652370 10.1007/s10654-010-9491-z

[11] Grossmann NC, Soria F, Juvet T, Potretzka A, Djaladat H, Kikuchi E, Mari A, Khene Z, Fujita K, Raman JD, et al. Comparing oncological and perioperative outcomes of open versus laparoscopic versus robotic radical nephroureterectomy for the treatment of upper tract urothelial carcinoma: A multicenter, multinational, propensity score-matched analysis. Eur Urol. 2023;83:S737–8. 10.3390/cancers15051409 PMC10000228 36900201

[12] Li C-C, Chang C-H, Huang C-P, Hong J-H, Huang C-Y, Chen IHA, et al. Comparing oncological outcomes and surgical complications of Hand-Assisted, laparoscopic and robotic nephroureterectomy for upper tract urothelial carcinoma. Front Oncol. 2021;11. 10.3389/fonc.2021.731460 PMC8522474 34671556

[13] Kim TH, Hong B, Seo HK, Kang SH, Ku JH, Jeong BC. The comparison of oncologic outcomes between open and laparoscopic radical nephroureterectomy for the treatment of upper tract urothelial carcinoma: A Korean multicenter collaborative study. Cancer Res Treat. 2019;51(1):240–51. 29690748 10.4143/crt.2017.417 PMC6333991

[14] Kim HS, Ku JH, Jeong CW, Kwak C, Kim HH. Laparoscopic radical nephroureterectomy is associated with worse survival outcomes than open radical nephroureterectomy in patients with locally advanced upper tract urothelial carcinoma. World J Urol. 2016;34(6):859–69. 26497823 10.1007/s00345-015-1712-3

[15] Liu J-Y, Dai Y-B, Zhou F-J, Long Z, Li Y-H, Xie D, et al. Laparoscopic versus open nephroureterectomy to treat localized and/or locally advanced upper tract urothelial carcinoma: oncological outcomes from a multicenter study. BMC Surg. 2017;17(1):8. 10.1186/s12893-016-0202-x. 10.1186/s12893-016-0202-x PMC5240226 28095848

[16] Kim SH, Song MK, Kim JK, Hong B, Kang SH, Ku JH, Jeong BC, Seo HK. Laparoscopy versus open nephroureterectomy in prognostic outcome of patients with advanced upper tract urothelial cancer: A retrospective, multicenter, Propensity-Score matching analysis. Cancer Res Treat. 2019;51(3):963–72. 30322230 10.4143/crt.2018.465 PMC6639211

[17] Kido K, Hatakeyama S, Fujita N, Yamamoto H, Tobisawa Y, Yoneyama T, Yoneyama T, Hashimoto Y, Koie T, Iwabuchi I, et al. Oncologic outcomes for open and laparoscopic radical nephroureterectomy in patients with upper tract urothelial carcinoma. Int J Clin Oncol. 2018;23(4):726–33. 29435873 10.1007/s10147-018-1248-9

[18] Clements MB, Krupski TL, Culp SH. Robotic-Assisted surgery for upper tract urothelial carcinoma: A comparative survival analysis. Ann Surg Oncol. 2018;25(9):2550–62. 29948423 10.1245/s10434-018-6557-8

[19] Hoeh B, Kosiba M, Wenzel M, Meister N, Preisser F, Shariat SF, Hohenhorst JL, Becker A, Mandel P, Banek S, et al. Comparison of survival outcomes between laparoscopic versus open radical nephroureterectomy in upper tract urothelial cancer patients: experiences of a tertiary care single center. Curr Urol. 2023;17(4):292–8. 37994335 10.1097/CU9.0000000000000113 PMC10662872

[20] Morselli S, Vitelli FD, Verrini G, Sebastianelli A, Campi R, Liaci A, Spatafora P, Barzaghi P, Ferrari G, Gacci M, et al. Comparison of tumor seeding and recurrence rate after laparoscopic vs. Open nephroureterectomy for upper urinary tract transitional cell carcinoma. Front Surg. 2021;8:769527. 35004836 10.3389/fsurg.2021.769527 PMC8732869

[21] Cui Qa, Qin M, Zhang W, Liu Q, Jiang S, Zhang J, Ding S, Dai K, Qin Z, Wang W, et al. Efficacy analysis of robot-assisted radical nephroureterectomy and laparoscopic radical nephroureterectomy for non-metastatic high-risk upper tract urothelial carcinoma. Jounral Chongqing Med Univ. 2022;47(4):439–43.

[22] Miyazaki J, Nishiyama H, Fujimoto H, Ohyama C, Koie T, Hinotsu S, Kikuchi E, Sakura M, Inokuchi J, Hara T, et al. Laparoscopic versus open nephroureterectomy in Muscle-Invasive upper tract urothelial carcinoma: subanalysis of the Multi-Institutional National database of the Japanese urological association. J Endourol. 2016;30(5):520–5. 26669358 10.1089/end.2015.0757

[23] Chung S-D, Chueh S-C, Lai M-K, Huang C-Y, Pu Y-S, Yu H-J, Huang K-H. Long-term outcome of hand-assisted laparoscopic radical nephroureterectomy for upper-tract urothelial carcinoma: comparison with open surgery. J Endourol. 2007;21(6):595–9. 17638552 10.1089/end.2006.9948

[24] Roupret M, Hupertan V, Sanderson KM, Harmon JD, Cathelineau X, Barret E, Vallancien G, Rozet F. Oncologic control after open or laparoscopic nephroureterectomy for upper urinary tract transitional cell carcinoma:: A single center experience. Urology. 2007;69(4):656–61. 17445646 10.1016/j.urology.2007.01.007

[25] Hu C-Y, Yang C-K, Huang C-Y, Ou Y-C, Hung S-F, Chung S-D, Pu Y-S. Robot-Assisted Laparoscopic Nephroureterectomy versus Hand-Assisted Laparoscopic Nephroureterectomy for Upper Urinary Tract Urothelial Carcinoma: A Matched Comparison Study. *Biomed Research International* 2015, 2015. 10.1155/2015/918486 PMC4619898 26539538

[26] Vasudeo V, Singh A, Khanna A, Rawal SK, Pratihar SK, Saurabh N, Kumar B, Ali M, Sharma P, Akotkar S, et al. Surgical and oncological outcomes of robot-assisted versus laparoscopic radical nephroureterectomy for upper-tract urothelial carcinoma: A single-center comparative analysis. Indian J Urology: IJU: J Urol Soc India. 2023;39(4):285–91. 10.4103/iju.iju_128_23 PMC10704978 38077196

[27] Fairey AS, Kassouf W, Estey E, Tanguay S, Rendon R, Bell D, Izawa J, Chin J, Kapoor A, Matsumoto E, et al. Comparison of oncological outcomes for open and laparoscopic radical nephroureterectomy: results from the Canadian upper tract collaboration. BJU Int. 2013;112(6):791–7. 23148712 10.1111/j.1464-410X.2012.11474.x

[28] Ni S, Tao W, Chen Q, Liu L, Jiang H, Hu H, Han R, Wang C. Laparoscopic versus open nephroureterectomy for the treatment of upper urinary tract urothelial carcinoma: a systematic review and cumulative analysis of comparative studies. Eur Urol. 2012;61(6):1142–53. 22349569 10.1016/j.eururo.2012.02.019

[29] Ariane MM, Colin P, Ouzzane A, Pignot G, Audouin M, Cornu J-N, Albouy B, Guillotreau J, Neuzillet Y, Crouzet S, et al. Assessment of oncologic control obtained after open versus laparoscopic nephroureterectomy for upper urinary tract urothelial carcinomas (UUT-UCs): results from a large French multicenter collaborative study. Ann Surg Oncol. 2012;19(1):301–8. 21691878 10.1245/s10434-011-1841-x

[30] Taylor BL, Scherr DS. Robotic nephroureterectomy. Urologic Clin North Am. 2018;45(2):189–97. 10.1016/j.ucl.2017.12.004 29650135

[31] Kenigsberg AP, Smith W, Meng X, Ghandour R, Rapoport L, Bagrodia A, Lotan Y, Woldu SL, Margulis V. Robotic nephroureterectomy vs laparoscopic nephroureterectomy: increased utilization, rates of lymphadenectomy, decreased morbidity robotically. J Endourol. 2021;35(3):312–8. 33081512 10.1089/end.2020.0496

[32] Wolf JS, Moon TD, Nakada SY. Hand assisted laparoscopic nephrectomy: comparison to standard laparoscopic nephrectomy. J Urol. 1998;160(1):22–7. 9628597

[33] Munver R, Del Pizzo JJ, Sosa RE. Hand-assisted laparoscopic nephroureterectomy for upper urinary-tract transitional-cell carcinoma. J Endourol. 2004;18(4):351–8. 15253785 10.1089/089277904323056898

[34] Kitamura H, Maeda T, Tanaka T, Fukuta F, Kobayashi K, Nishiyama N, Takahashi S, Masumori N. Comparison of laparoscopic, hand-assisted, and open surgical nephroureterectomy. JSLS: J Soc Laparoendoscopic Surg. 2014;18(2):288–93. 10.4293/108680813X13794522666842 PMC4035642 24960495

[35] Nouralizadeh A, Tabatabaei S, Basiri A, Simforoosh N, Soleimani M, Javanmard B, Ansari A, Shemshaki H. Comparison of open versus laparoscopic versus Hand-Assisted laparoscopic nephroureterectomy: A systematic review and Meta-Analysis. J Laparoendoscopic Adv Surg Techniques Part A. 2018;28(6):656–81. 10.1089/lap.2017.0662 29461914

[36] Brouwer OR, Albersen M, Parnham A, Protzel C, Pettaway CA, Ayres B, Antunes-Lopes T, Barreto L, Campi R, Crook J, et al. European association of Urology-American society of clinical oncology collaborative guideline on penile cancer: 2023 update. Eur Urol. 2023;83(6):548–60. 36906413 10.1016/j.eururo.2023.02.027

